# TCCbuilder: An open-source tool for the analysis of thermal switches, thermal diodes, thermal regulators, and thermal control circuits

**DOI:** 10.1016/j.isci.2024.111263

**Published:** 2024-10-28

**Authors:** Katja Vozel, Katja Klinar, Nada Petelin, Andrej Kitanovski

**Affiliations:** 1 Faculty of Mechanical Engineering, University of Ljubljana, Askerceva 6, 1000 Ljubljana, Slovenia

**Keywords:** Heat transfer, Open source software, Thermal engineering, Thermal property

## Abstract

In the area of thermal management, thermal control elements (TCEs) and thermal control circuits (TCCs) are proving to be innovative solutions to the challenges of temperature control and heat dissipation in various applications, ranging from electronic cooling to energy conversion and temperature control in buildings. Their integration promises to improve power density, energy efficiency, system reliability and system life expectancy. With the aim of connecting researchers in the field of thermal management and accelerating the development of TCEs and TCCs, we have developed an open-source TCC simulation tool called TCCbuilder that enables a quick and easy time-dependent 1D numerical analysis of the behavior of TCEs and TCCs. It uses the heat conduction equation to solve temperature profiles in different devices. The TCCbuilder application offers features not previously available with any other TCC modeling tool: a large adjacent library of materials and TCEs as well as a user-friendly graphical user interface (GUI).

## Highlights

• An open-source tool for modeling thermal control elements and circuits has been built
• The tool contains a comprehensive library of materials and a user-friendly GUI
• It offers a range of components, such as thermal switches and thermal diodes
• Its capabilities were demonstrated using various application examples

## Graphical abstract

**Figure:**
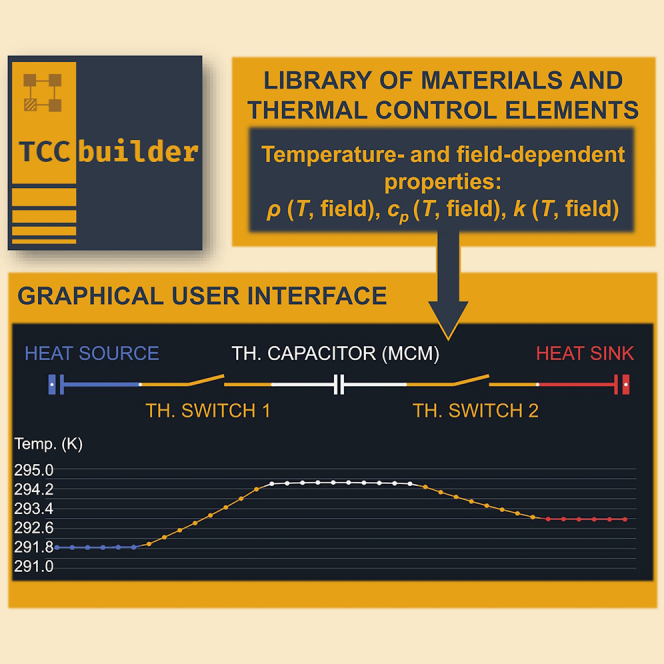

## Introduction

**Figure 1:**
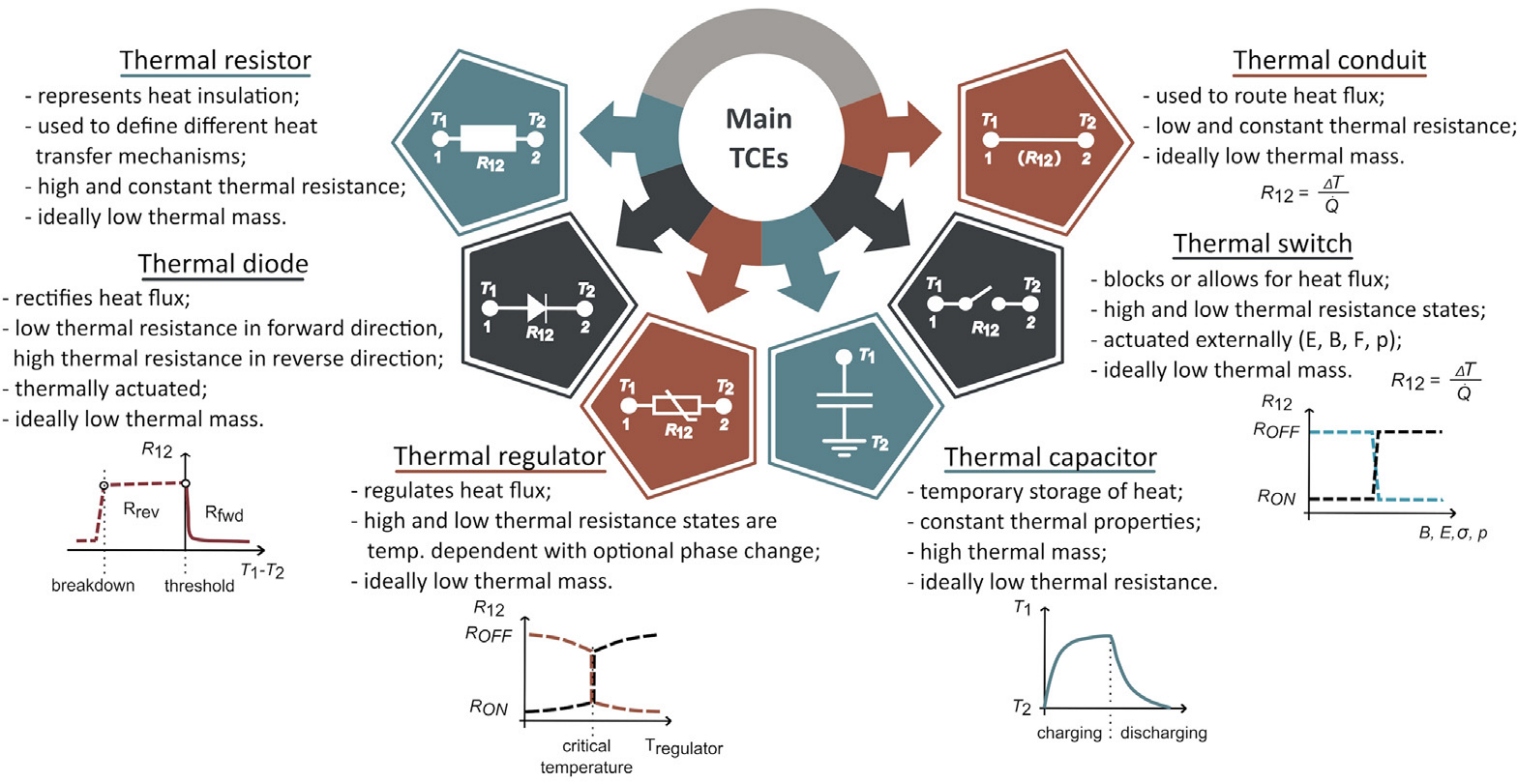
Six main TCEs with their properties and operational characteristics

The increasing demand for better operational and energy efficiency of systems, especially small electronic systems with a high energy density, requires better thermal management. Thermal control elements (TCEs) and thermal control circuits (TCCs) are a promising addition to conventional thermal management methods. They can be very useful when solving the issues of thermal management in applications like cryogenics,^1^^,^^2^^,^^3^ energy conversion,^4^^,^^5^ microfluidics,^6^^,^^7^ biology, chemistry, and pharmacy,^8^^,^^9^^,^^10^ buildings,^11^^,^^12^^,^^13^ outer space,^14^^,^^15^ sensors,^16^^,^^17^ and caloric cooling.^18^^,^^19^^,^^20^ TCEs, similar to their electrical counterparts, provide a nonlinear, switchable, and active control of heat.^21^^,^^22^^,^^23^ They include thermal conduits, thermal resistors, thermal switches, thermal regulators, thermal diodes, and thermal capacitors. Thermal conduits represent solid-state routers for heat; thermal resistors are used for thermal insulation; thermal switches actively control heat transfer by switching between states of high thermal resistance and low thermal resistance; thermal regulators and capacitors can maintain a desired temperature; and thermal diodes can rectify heat flux. Figure 1 explains the characteristics of TCEs. Each TCE controls the heat transfer between two terminals with temperatures $T_{1}$ and $T_{2}$, either with a constant or a variable thermal resistance $R_{12}$. For the thermal switch and the thermal regulator, $R_{12}=R_{\text{ON}}$ in the low thermal resistance state and $R_{12}=R_{\text{OFF}}$ in the high thermal resistance state. Thermal switches are actuated with an external field that is either magnetic with a magnetic field density *B*, electric with a strength *E*, stress with a magnitude σ, or pressure with a magnitude *p*. Thermal regulators are not actuated externally, but change their conductance from low to high or vice versa at a critical temperature. Consequently, thermal switches have a control-dependent linear transfer function, while the transfer function of thermal regulators is strongly nonlinear with temperature.^21^ For the thermal diode, $R_{12}=R_{\text{fwd}}$ in the forward direction and $R_{21}=R_{\text{rev}}$ in the reverse direction. The conductance of thermal diodes depends on the direction of the temperature gradient in relation to the orientation of the thermal diode.

However, with a few exceptions in cryogenics,^24^ TCEs and TCCs remain at an early stage of development. In recent years, there have been many publications dealing with TCEs, including several reviews.^21^^,^^22^^,^^23^^,^^25^^,^^26^ Most papers on TCEs focus on a single TCE and tend to search for suitable materials and geometries to achieve better performance. Among these, we should mention that there have been many improvements in the field of thermal switches, which are one of the most important thermal control elements since their switching ratio (SR) generally far exceeds the rectification ratio of thermal diodes and is able to allow or stop heat flow regardless of the direction of the temperature gradient. For example, in ferroelectric materials, the application of an electric field can strongly change the conductivity of the material. Liu et al.^27^ calculated an SR of 1.8 in PbTiO3 at room temperature considering an electric field of 2000 kVcm^−1^. Further calculations by Liu et al.^28^ showed an SR of 5.8 in some ferroelectric materials at room temperature using an electric field of 4000 kVcm^−1^. For antiferroelectric lead(II) zirconate (PbZrO3) materials, an SR of 2.3 was calculated and experimentally verified at room temperature and an electric field of only 600 kVcm^−1^.^29^

While searching for suitable materials and focusing on specific TCEs is an important aspect and the research needs to continue, there are also some concepts, such as combining TCEs in thermal control circuits (TCCs) and the transient operation of TCEs and TCCs, that have not yet been properly analyzed. For example, there is no comprehensive analysis of which TCCs resembling electronic circuits are possible and what the minimum effectiveness of the TCEs forming them must be for them to function properly. In addition, the study of transient operation is often limited to one kind of periodic boundary condition or one kind of dependence of thermal conductivity and heat capacity on temperature.^30^^,^^31^

The key to the development of TCEs and TCCs is the virtual design and numerical modeling of their behavior. It is crucial to be able to quickly validate new concepts, test a large number of cases and optimize parameters. So far, several tools offer the three-dimensional modeling of heat transfer, e.g., Comsol,^32^ Ansys,^33^ SimScale,^34^ and OpenFoam.^35^ However, these are multi-purpose products with a complex calculation infrastructure that, with the exception of OpenFoam, are not open source. To use any of these products, the user needs considerable knowledge of numerical modeling and a certain amount of training. In addition, these products are not specialized in the design of the TCCs and have only a limited database of relevant temperature- and external field-dependent properties (e.g., density, specific heat capacity and thermal conductivity) of various materials suitable for the design of TCCs. There is also a lack of models for certain applications with cyclic operation, e.g., for caloric devices, where TCEs could be indispensable as they would replace an active caloric regenerator (using a working fluid).^18^ This would lead to faster and more efficient heat transfer between the caloric material and other parts of a device, which in turn would improve its power density and energy efficiency.^18^^,^^36^

In 2018, Silva et al. published an open-source Python framework called Heatrapy^37^ that is dedicated to time-dependent (transient) 1D and 2D heat-transfer modeling and focuses on the modeling of caloric effects. The Heatrapy framework has a limited database of materials with characterized properties (density, specific heat capacity, and thermal conductivity).

As a next step, we have developed an open-source TCC simulation tool called TCCbuilder, which focuses on the design of TCEs and TCCs for any macroscopic application. TCCbuilder enables the quick and easy analysis of the behavior of TCEs and TCCs in (quasi-)steady-state and transient operation. It is based on a 1D numerical model for heat conduction to solve temperature profiles in the modeled structures. This should lead to the faster development of thermal management systems to improve their power density, energy efficiency, reliability and life expectancy. The TCCbuilder application offers features not previously offered by any other TCC modeling tool: a large adjacent library of materials suitable for designing TCEs and characterized for use in TCCbuilder, a library of already designed TCEs from the literature, and a user-friendly graphical user interface (GUI). The platform will also serve as a connection center for thermal management researchers around the world who will collaboratively improve and expand the tool.

Table 1 provides an overview of the main heat-transfer modeling tools currently available.

**Table 1:** Comparison of heat-transfer modeling tools

	Comsol	Ansys	SimScale	OpenFoam	Heatrapy	TCCbuilder
Focus	General	General	General	Computational fluid dynamics	2D heat-transfer modeling, calorics	1D heat-transfer modeling, TCE and TCC design
Open-source	No	No	No	Yes	Yes	Yes
Required level of knowledge of num. modeling	Medium	Medium	Medium	High	Low	Low
Computational complexity	High	High	High	High	Medium	Low
Library of materials for TCEs	No	No	No	No	No	Yes
Has a GUI	Yes	Yes	Yes	Yes	No	Yes

**Figure 2:**
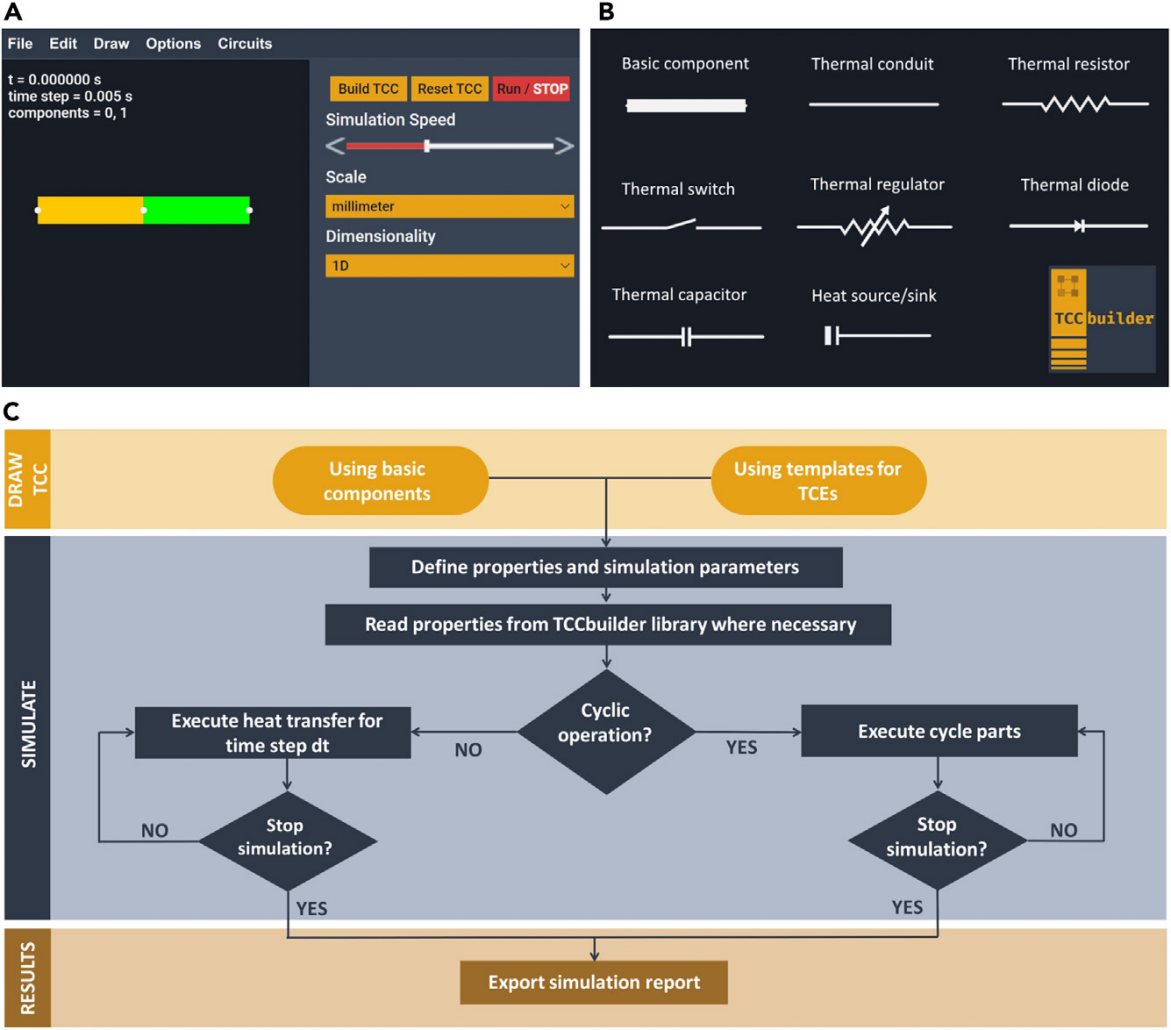
GUI of TCCbuilder (A) The menu, controls, and the canvas of the GUI, with a user-drawn solid-state thermal diode. (B) The basic component, the six main TCEs, and a heat source/sink, as represented in the GUI. (C) Flowchart of the modeling of TCCs in TCCbuilder. First, the user draws a TCC on the canvas, specifies the materials and properties for the components or TCEs, sets the simulation parameters, and starts the simulation. The simulation runs until the user stops it.

The GUI of TCCbuilder is available online as a web application.^38^ Figure 2A shows the appearance of the application, with an example of a user-drawn solid-state thermal diode. The user can draw any TCC on the canvas using basic components or the available TCEs (Figure 2B), and specify their properties or materials. Then, they can set the simulation parameters and run a simulation. The flowchart of the modeling of TCCs in TCCbuilder is shown in Figure 2C.

## Results

### Library of materials and TCEs

The TCCbuilder’s library of materials and TCEs is the result of an extensive literature review and serves as the basis for the input parameters in TCCbuilder. It is divided into two, constantly expanded and improved open-source repositories: one for the materials,^39^ and one for the TCEs.^40^

The library of materials can help a user to quickly evaluate a TCE or TCC, even if they do not have their own data for the properties of the materials being used. Each material is assigned an ID depending on its classification and dimensionality (bulk, film, or 1D). We have divided the materials into six groups: Metals and Alloys, Ceramics, Polymers, Composites, Semiconductors, and Biomaterials. In general, the library contains information about the temperature dependence of the properties: density, specific heat capacity, and thermal conductivity, but also emissivity and melting point. Other information includes, for example, for caloric materials, the external magnetic, electric, stress, or pressure field strengths at which the adiabatic temperature change is specified, the adiabatic temperature change as a function of the external field strength and the temperature, the specific heat capacity as a function of the external field strength and the temperature, and the hysteresis of a material. The library of materials also has its own GUI, which allows the user to display and compare the data for different materials.^41^

The library of TCEs enables the user to select already-designed and tested TCEs in their own TCC. The TCEs from the library are available directly in the GUI via the “Draw” menu. Depending on the type, each TCE is represented by one of the TCE representations: as a thermal switch, thermal regulator or thermal diode (Figure 2A). The properties of each TCE from the library are defined to be as similar as possible to the device from the source paper and are often given as effective values corresponding to a 1D representation of the TCE. For this reason, the TCEs from the library often cannot be resized and their operating temperature range is given to inform the user at what temperatures the device has been evaluated.

### Modeling results

In this section we present several examples of using TCCbuilder to analyze TCEs or TCCs in steady-state and transient operation. Some of the examples were also used to validate the tool.

#### Example 1: TCCbuilder validation with a solid-state thermal diode

Kobayashi et al.^42^ fabricated a solid-state thermal diode consisting of two components: one of LSCO (La_0.7_ Sr_0.3_ CoO_3_) material and the other of LCO (LaCoO_3_) material (Figure 2A). In the selected operating range of the diode at low temperatures from 40 to 100 K, the thermal conductivity of LSCO increases with temperature and the thermal conductivity of LCO decreases with temperature. When the LSCO end of the diode is at a higher temperature and the LCO end is at a lower temperature, the thermal conductivities of both materials have the highest values. Therefore, the heat flux from the hot end to the cold end is greater in this forward case than in the reverse case, when the LSCO part has a lower temperature than the LCO part of the diode. The diode parameters and the boundary conditions for the experiment were as follows^42^: LSCO length = 6.1 mm, LCO length = 6.3 mm, the temperature of the cold side was kept at 40 K. A rectification factor, defined as the ratio of heat fluxes in the forward and reverse directions, of 1.43 was measured at ΔT= 58.9 K (the difference in the temperatures at both ends of the diode). The measured heat fluxes in the forward and reverse directions in the steady state were 13,180 Wm^–2^ and 9,240 Wm^–2^. The calculated values were 11,010 Wm^–2^ and 7,515 Wm^–2^, giving a rectification factor of 1.47.^42^

Using the same parameters in TCCbuilder, we obtained a rectification factor of 1.44 in the steady state, with corresponding heat fluxes of 11,090 Wm^–2^ and 7,687 Wm^–2^. Constant-temperature boundary conditions, 10 control volumes (CVs) for each component, and a time step of 10 ms were used. It took about 100 s to reach the steady state from the initial temperature of 50 K, with a calculation time of 5 s. The results in TCCbuilder agree reasonably well with the results from^42^; therefore, the simulation can validate the tool in the steady state.

#### Example 2: Modeling the transient operation of a TCC in electronics

**Figure 3:**
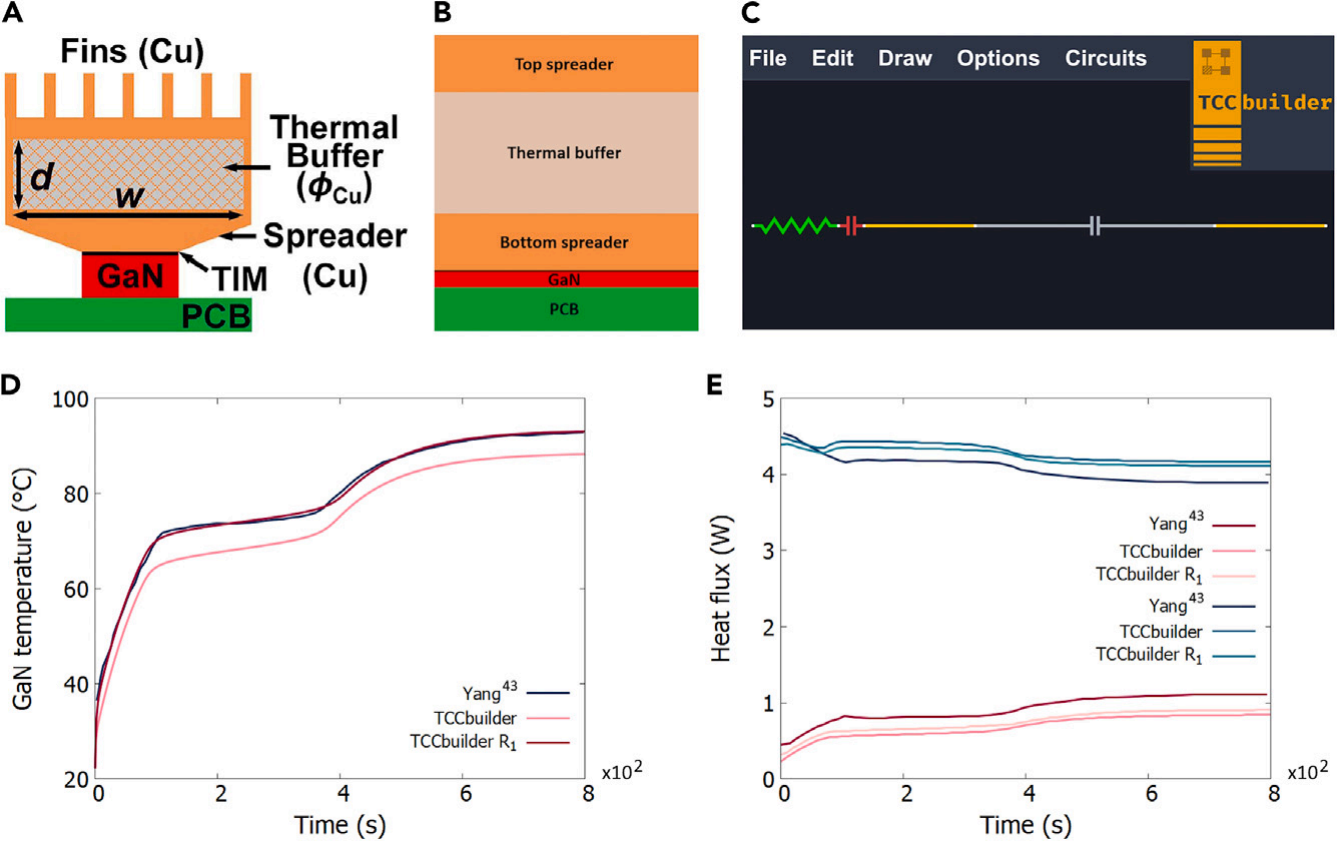
System for cooling a GaN device placed on a printed-circuit board and the temporal development of the GaN temperature and the heat fluxes inside the TCC (A) A 2D representation. Adapted with permission from.^43^ (B) An equivalent 1D TCC as used in TCCbuilder. (C) The TCC as represented in TCCbuilder, from left to right: PCB as a thermal resistor, GaN device as a thermal capacitor, bottom thermal spreader as a thermal conduit, thermal buffer with PCM as a thermal capacitor, and top thermal spreader as a thermal conduit. The TCC is rotated by 90° with respect to (B) so that the direction of the heat flux is horizontal. (D) Temperature as measured and simulated in^43^ (blue line) and calculated with TCCbuilder (dark-pink line). The light-pink line represents the values calculated with TCCbuilder when the thermal contact resistance between the GaN and the bottom spreader is twice as high as in the experiment. (E) Comparison of the temporal development of the heat fluxes from the GaN device to the bottom and to the top of the TCC, as measured and simulated in^43^ (red and dark-blue line) and calculated with TCCbuilder (dark-pink and blue line). The light-pink and cyan lines represent the values calculated with TCCbuilder when the thermal contact resistance between the GaN and the bottom spreader is twice as high as reported in the experiment.

An example of a TCC is taken from.^43^ The TCC represents a system for the cooling of a microelectronic gallium nitride (GaN) device placed on a printed-circuit board (PCB). The GaN device is cooled via a thermal buffer consisting of a phase-change material (PCM). There are copper fins on the top of the thermal buffer, which improve heat transfer to the environment through forced convection. Figure 3A shows the basic components of the system: a PCB, a GaN device, a thermal spreader, a thermal buffer, and a top thermal spreader with fins.

A computer-aided, finite-element-analysis simulation for the aforementioned system was performed in COMSOL Multiphysics.^32^^,^^43^ To model the system in TCCbuilder, we created an almost equivalent 1D TCC, assuming a cross-sectional area of the thermal buffer A= 2.56 cm^−2^ (Figure 3B) for the heat flux.

Figure 3D shows the temporal development of the GaN temperature of both the ref.^43^ and the TCCbuilder. The difference between the temperatures is about 6 K, which accounts for about 8% of the total temperature rise of the GaN. However, increasing the thermal resistance between the GaN and the bottom spreader from $R_{1}=$ 7.0 × 10^−4^ m^2^ kW^−1^ to $R_{2}=$ 1.4 × 10^−3^ m^2^ kW^−1^ results in a match (red line in Figure 3A). Figure 3E shows a comparison of the heat fluxes calculated in^43^ and in TCCbuilder.

#### Example 3: Modeling of a double-unit electrocaloric cooling device

Bo et al.^44^ demonstrated the operation of a polymer-based, double-unit electrocaloric (EC) cooling device. The cascading of EC devices is a way to increase the temperature span, which is necessary for the improved performance of cooling devices.^45^

**Figure 4:**
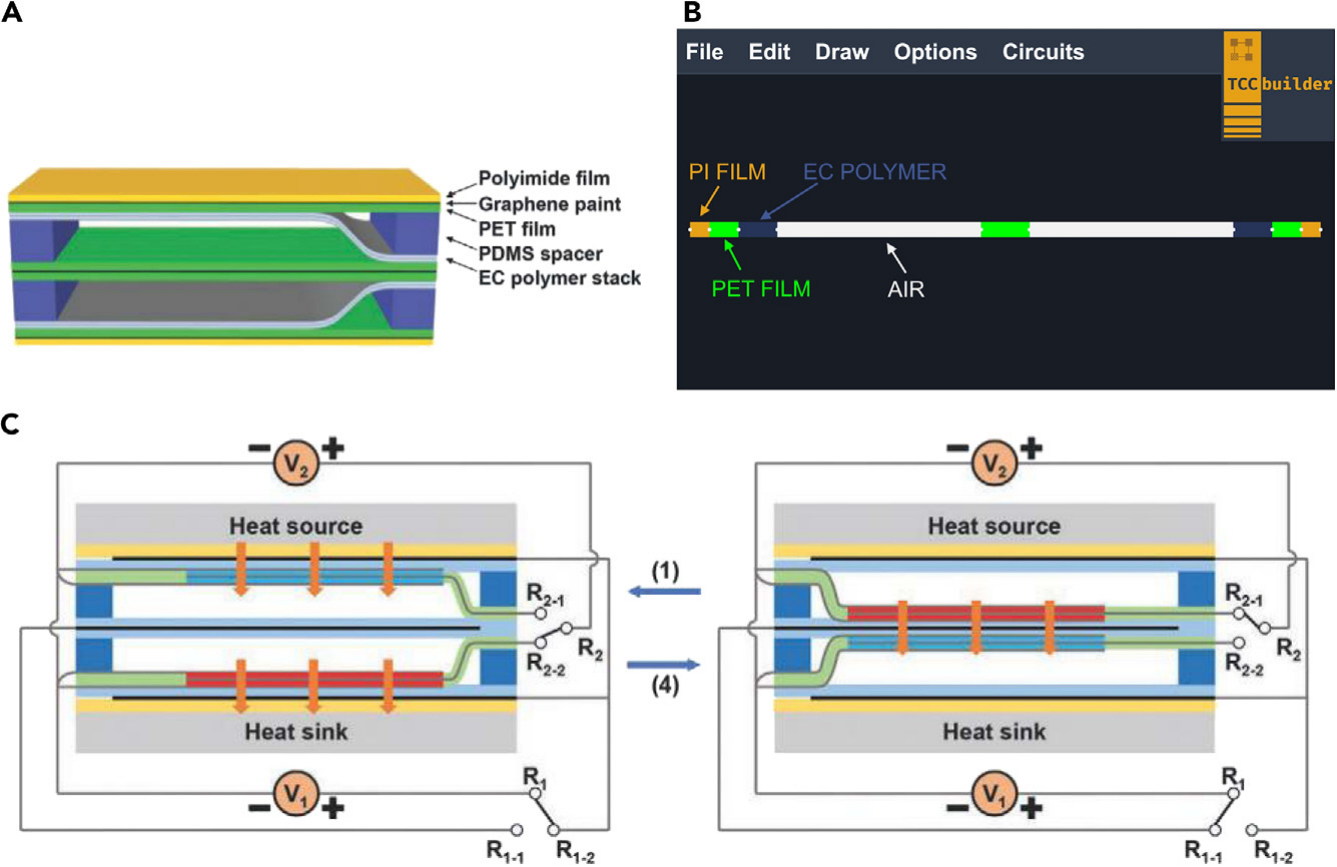
Double-unit electrocaloric (EC) polymer-based cooling device (A) The composition of the device. Adapted with permission from.^44^ (B) The equivalent 1D TCC of a double-unit EC cooling device. In the figure, the direction of heat flux is only vertical. (C) Operation of the device. The blue and red colors of the EC polymers represent their temperature and the orange arrows show the direction of the heat flux. Adapted with permission from.^44^

Figure 4A is a schematic of a double-unit cooling device based on two-layer EC polymer stacks. For a detailed explanation of the composition and operation of the device, refer to.^44^Figure 4C illustrates the operation of the double-unit EC device. The six phases of operation are: (1) The EC polymers contact the heat source and heat sink of the device, and the electric field is turned off for the upper polymer, causing the temperature of the upper polymer to decrease due to the electrocaloric effect, and applied to the lower polymer, causing the temperature of the lower polymer to increase due to the electrocaloric effect. (2) Heat is transferred from the heat source to the upper polymer and from the lower polymer to the heat sink. (3) The polymers are moved by an electrostatic field to the common graphene electrode in the center of the device. (4) An electric field is turned off for the lower polymer, resulting in a temperature decrease of the lower polymer due to the electrocaloric effect, and applied to the upper polymer, resulting in a temperature increase of the upper polymer due to the electrocaloric effect. (5) Heat is transferred from the upper polymer to the lower polymer. (6) The polymers are moved by electrostatic fields to the heat sink and the heat source so that the cycle can repeat. In this way, heat is efficiently transferred from the heat source to the heat sink.

**Figure 5:**
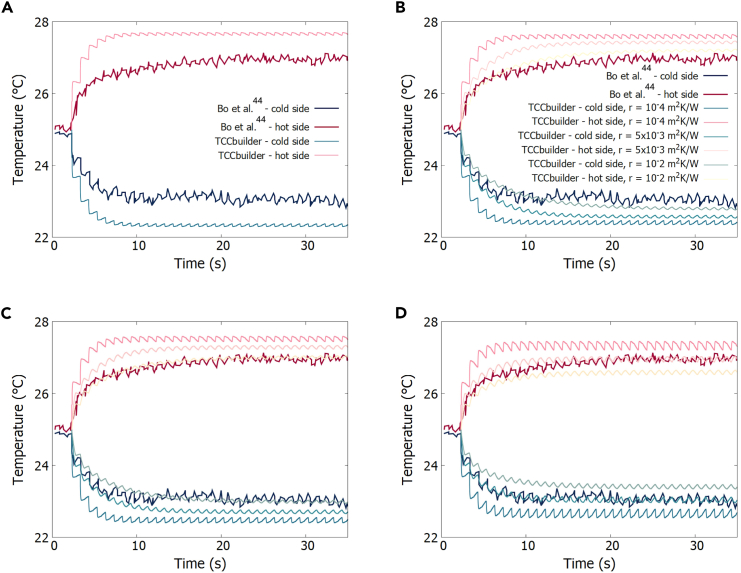
Comparison of the temporal development of a temperature span of the double-unit electrocaloric (EC) polymer-based cooling device operating with an electric field of 60.6 MVm^–1^ and a frequency of 1 Hz, as obtained from the experiment and calculated with TCCbuilder (A) Idealized case with h= 0 Wm^–2^ K^−1^ and R= 0 m^2^ kW^−1^. (B) With the convection coefficient set to h= 5 Wm^–2^ K^−1^ and different values of *R*. (C) With the convection coefficient set to h= 10 Wm^–2^ K^−1^ and different values of *R*. (D) With the convection coefficient set to h= 25 Wm^–2^ K^−1^ and different values of *R*. The legend in (B) also applies for (C) and (D).

Figure 4B shows the equivalent 1D TCC in TCCbuilder. Since the thermal contact resistances and the value of the convection coefficient are not known, we initially simulate the device when these two values are set to zero. This corresponds to an ideal case where the temperature span of the device is almost twice the adiabatic temperature change of the EC polymer. Figure 5A shows the temperatures on both sides of the double-unit device as measured in the experiment^44^ and calculated in TCCbuilder with h= 0 Wm^–2^ K^−1^ and R=0 m^2^ KW^−1^. If no losses are taken into account, TCCbuilder overestimates the temperature span of the device (ΔT= 5.4 K), which is close to $2\times \left|\Delta T_{\text{ad}}\right|=$ 5.6 K of the EC polymer.

Figures 5B–5D show a comparison between the experiment and the TCCbuilder simulation with different values of *R* and for different values of the convection coefficient, *h* = 5, 10 or 25 Wm^–2^ K^−1^. The results of the experiment fall within the results of the simulations. From this we can conclude that the actual values of the thermal contact resistance and the convection coefficient are located somewhere in the predicted ranges.

#### Example 4: TCCbuilder validation with a TCC comprising a heat engine

Zhang et al.^46^ investigated a thermal control circuit for energy harvesting from ambient thermal fluctuations:

(Equation 1) $${\tilde{T}}_{\text{amb}}\left(t\right)=T_{0}+T_{A}\;\sin\left(\omega t\right)\text{,}$$

where $T_{0}$ is the median ambient temperature, $T_{A}$ is the amplitude of fluctuation, ω is the frequency of fluctuation and *t* stands for time.

**Figure 6:**
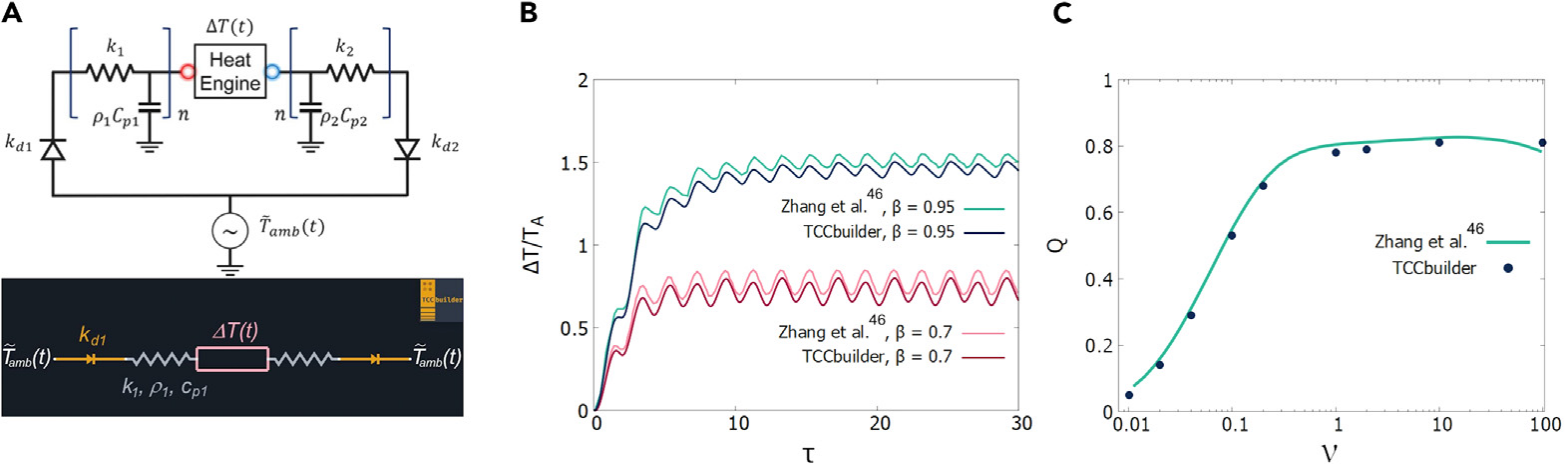
TCC for energy harvesting from ambient thermal fluctuations (A) Top: TCC for energy harvesting from ambient-temperature fluctuations, adapted with permission from.^46^ Bottom: TCC representation in TCCbuilder. (B) Comparison of temporal development of dimensionless temperature difference across the heat engine as obtained in^46^ and calculated with TCCbuilder, for different β parameters of the thermal diodes. (C) Comparison of dimensionless power of the heat engine as given in^46^ and calculated with TCCbuilder, with respect to the dimensionless frequency ν of the ambient-temperature fluctuation.

The TCC consists of a thermoelectric (TE) heat engine, two thermal masses (each thermal mass is represented by a thermal capacitor having a negligible thermal resistance and a thermal resistor having negligible thermal mass) and two thermal diodes, as shown in the top part of Figure 6A. Such a configuration of TCEs creates a large single-polarity temperature difference across the heat engine to enable optimal energy harvesting. In TCCbuilder, only the diodes and the resistors are used, because the density and specific heat capacity of each thermal mass are added to the resistors (bottom of Figure 6A.), corresponding to the original configuration. The TCC is opened in a straight line with the ambient-temperature variation as a boundary condition on both sides.

The diodes used in the TCC are identical and have a theoretical thermal conductivity function $k_{d}\left(T_{w},T_{e}\right)$, described by

(Equation 2) $$k_{d}=k_{d,0}\left(1+\frac{2\beta }{\pi }\arctan\left(\gamma_{d}\left(T_{w}-T_{e}\right)\right)\right)\text{,}$$

where β and $\gamma_{d}$ are related to the level of thermal rectification and the steepness in the change of the thermal conductivity of the thermal diode, $k_{d,0}$ is the median thermal conductivity of the thermal diode, and $T_{w}$, $T_{e}$ are the temperatures at the left and right ends of a diode (at the interface of a diode and the ambient or at the interface of a diode and a linear thermal mass). The diode with such a temperature dependence of thermal conductivity is added to the TCCbuilder as an example of a theoretically described diode.

Figure 6B shows a comparison of the temperature differences across the heat engine, as reported in^46^ and calculated with TCCbuilder for β = 0.7 and β = 0.95.

The power density of the TE heat engine is defined in dimensionless form by the power metric *Q*, which is defined as the time-averaged square of the temperature difference ΔT across the heat engine, normalized by the maximum-achievable temperature difference (2 $T_{A}$):

(Equation 3) $$Q=\frac{\bar{\Delta T^{2}}}{4T_{A}^{2}}\text{.}$$

This is added as an output to the code of the TE heat engine in TCCbuilder. Figure 6C shows a comparison of *Q*, as given in^46^ and calculated with TCCbuilder, with respect to the dimensionless frequency ν of the ambient-temperature fluctuation, for the case where β= 0.99.

#### Example 5: Validation in transient operation with a caloric device

The numerical model of TCCbuilder was also previously validated^47^ with the Heatrapy numerical model by Silva et al.^37^^,^^48^

**Figure 7:**
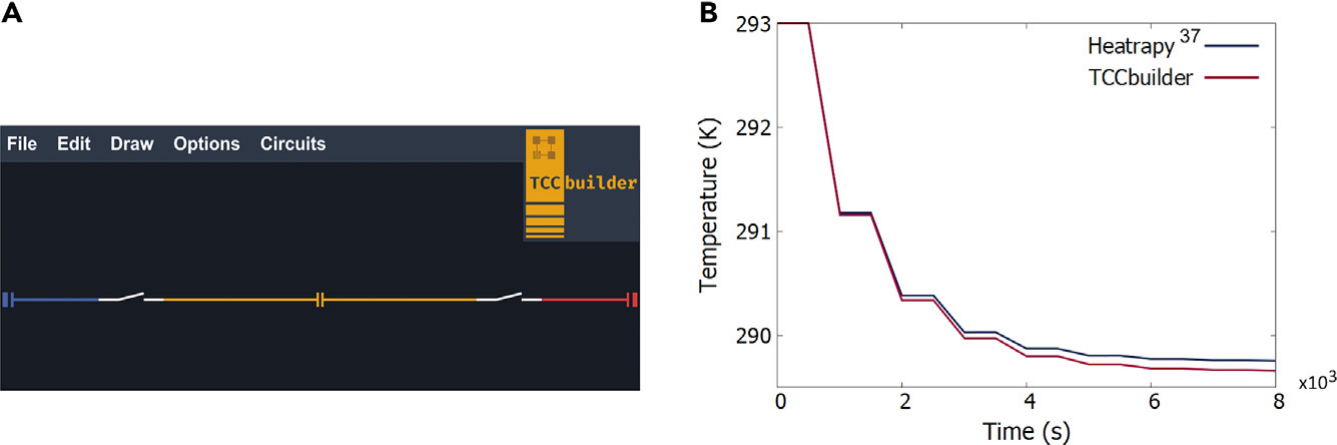
Validation of TCCbuilder with a magnetocaloric cooling device (A) A magnetocaloric cooling device, as represented in the TCCbuilder GUI, from left to right: heat source, thermal switch 1, magnetocaloric material, thermal switch 2, and heat sink. (B) Comparison of the temporal development of the heat-source temperature of a magnetocaloric cooling device operating with a magnetic field of 1 T and a frequency of 0.001 Hz, calculated with Heatrapy^37^ (blue line) and with TCCbuilder (red line).

Here we conduct the validation using the TCCbuilder GUI. The TCC used for the validation is a magnetocaloric device consisting of a magnetocaloric material (mcm), a heat source and a heat sink, and two thermal switches that control the heat transfer between the mcm and the heat source/sink (see Figure 7A). The device works with the thermodynamic Brayton cycle. When a magnetic field is applied to the mcm, the mcm heats up and the heat is transferred from the mcm to the heat source. The magnetic field is then removed from the mcm, causing it to cool down so that the heat can be transferred from the heat source to the mcm. The four processes of thermodynamic cycle (magnetization, demagnetization, and both heat transfer processes) are repeated cyclically. With an adiabatic boundary condition at the edge of the heat source, the temperature of the heat source decreases until a steady-state is reached. The parameters of the simulation can be found in the supplemental information of.^47^

Figure 7 shows the temporal development of the heat-source temperatures calculated with Heatrapy and with TCCbuilder. The temperature of the heat sink (not shown) is constant at 293 K due to the adiabatic boundary condition on the side of the heat sink. The temperature of the heat source decreases due to the magnetocaloric Brayton cycle. The boundary condition on the side of the heat source is convective with h= 10^6^ Wm^−2^ K^−1^. The quasi-steady-state temperature of the heat source in Heatrapy was 289.75 K and 289.66 K in TCCbuilder.

## Discussion

We have developed an open-source tool called TCCbuilder for the creation, analysis, and optimization of TCEs and TCCs. TCCbuilder works on a small scale from a few micrometers to a few tens of centimeters, considering heat conduction as the main heat-transfer mechanism. TCCbuilder has a user-friendly GUI with a large selection of different TCEs and an adjacent library of materials and TCEs. The library provides the properties (density, specific heat capacity, thermal conductivity, emissivity, and melting point) of various materials and the characteristics of some already built and tested TCEs. In addition to thermal conduction, TCCbuilder also includes caloric effects and the possibility of periodic (cyclic) operation with a wide range of cycle actions. TCCbuilder is the first tool to offer all of these features.

The tool was validated using examples from the literature, as shown in the main part of this paper: a solid-state thermal diode, energy harvesting from ambient-temperature fluctuations, and the transient operation of a caloric device. Examples are also given for the modeling of an electrocaloric cascade device and the transient operation of a thermal circuit for electronic cooling.

Numerous extensions are planned for TCCbuilder in the near future: The 1D part of TCCbuilder will be extended to include thermal radiation and convection in fluids. Further options for cycle parts, additional options for time-dependent boundary conditions and more operating characteristics of TCEs will be added. A separate model will be developed for the combination of TCEs and TCCs, where TCEs can be connected not only in series but also in parallel, allowing, among other things, the testing of more TCCs that resemble electronic circuits. A 2D heat-transfer model will be added and the library of materials and TCEs will be updated regularly.

The vision of TCCbuilder is to provide an important bridge between fundamental research and the development of systems that can later be tested and used to build real systems in the areas of cryogenics, energy conversion, microfluidics, biology, chemistry and pharmaceuticals, buildings, space, sensor technology, and caloric cooling. It will also serve as a solid foundation for knowledge exchange between fellow researchers in the fields of thermal management and materials. Researchers around the world are invited to contribute to the project, whether to improve the functionality or to add TCEs and materials to the library.

### Limitations of the study

Currently, the TCCbuilder model only considers macroscopic heat conduction. We will work on extensions to include other heat transfer processes and phenomena. Although the TCCbuilder model is 2D, the TCCbuilder online application can only handle 1D modeling of TCEs and TCCs.

## Resource availability

### Lead contact

Further information and requests for resources should be directed to, and will be fulfilled by the lead contact, K.K. (katja.klinar@fs.uni-lj.si).

### Materials availability

This study did not generate new unique materials.

### Data and code availability

• The material data has been deposited at Zenodo and is publicly available as of the date of publication. The DOIs of TCCbuilder’s library of materials and the library of thermal control elements are listed in the key resources table.
• The source code has been deposited at Zenodo and is publicly available as of the date of publication. The DOIs are listed in the key resources table.
• Any additional information required to analyze the data reported in this study is available from the lead contact upon request.

## Acknowledgments

The authors acknowledge the financial support of the 10.13039/501100004329Slovenian Research and Innovation Agency for the project TCCbuilder: An open-source simulation tool for thermal control circuits J7-3148, and the research core funding no. P2-0223.

## Author contributions

Conceptualization, K.K. and A.K.; methodology, K.V., K.K., N.P., and A.K.; investigation K.V., K.K., and N.P.; data curation, K.V.; visualization, K.V.; formal analysis, K.V.; writing—original draft, K.V.; writing—review and editing, K.K., N.P., and A.K.; funding acquisition A.K.; supervision, K.K. and A.K.

## Declaration of interests

A.K. is a member of iScience’s Advisory board and one of the Guest Editors of the Special Issue "Advanced thermal control: fundamentals and applications". The authors declare no competing interests.

## STAR★Methods

### Key resources table

**Table:** 

REAGENT or RESOURCE	SOURCE	IDENTIFIER
**Deposited data**		

TCCbuilder Library of Materials	LAHDE, University of Ljubljana	https://doi.org/10.5281/zenodo.13142749
TCCbuilder Library of TCEs	LAHDE, University of Ljubljana	https://doi.org/10.5281/zenodo.13219199
TCCbuilder source code	LAHDE, University of Ljubljana	https://doi.org/10.5281/zenodo.13142624

**Software and algorithms**		

Java	Oracle	https://www.java.com/
Google Web Toolkit	Google	https://www.gwtproject.org/
Circuit JS	Falstad	https://www.falstad.com/circuit/
TCCbuilderApp	LAHDE, University of Ljubljana	https://www.app.tccbuilder.org/

### Method details

#### The heat-transfer equation of the numerical model

The governing equation of the model is

(Equation 4) $$\rho c_{p}\frac{\partial T}{\partial t}=\nabla \left(k\nabla T\right)+\dot{q}\text{,}$$

where *T* stands for the temperature, *t* for the time, ∇ represents the space gradients, ρ stands for the density, $c_{p}$ stands for the specific heat capacity, k is the thermal conductivity tensor, and $\dot{q}$ stands for the other heat sources (for example, internal heat generation). In general, the properties ρ, $c_{p}$, and *k* are considered to be temperature and external field-dependent. In that case, they are read from the library of materials. The library contains the data of the properties in a certain temperature range (which is specified for each material) with an accuracy of 0.1 K. The value at the corresponding temperature is used, which avoids interpolation at runtime to make the calculation faster. Equation 4 is discretized in one dimension, so that, for each CV,

(Equation 5) $$\rho c_{p}\frac{T-T^{t-\Delta t}}{\Delta t}=\frac{1}{\Delta x}\left(k_{w}\frac{T_{w}-T}{\Delta {\tilde{x}}_{w}}-k_{e}\frac{T-T_{e}}{\Delta {\tilde{x}}_{e}}\right)+\dot{q}\text{.}$$

Here, $T=T_{n}$ is the temperature of the considered CV, with *x*-index *n*, and $T^{t-\Delta t}$ is the temperature of the same CV in the previous time step. The variable Δx represents the length of the considered CV. Subscripts w and e denote the west and east neighbours; $T_{w}=T_{n-1}$, $T_{e}=T_{n+1}$. The distance $\Delta {\tilde{x}}_{d}$ (d ∈ {w, e}) is the distance between the centers of neighbouring CVs and is equal to the sum of the half-lengths of neighbouring CVs: $\Delta {\tilde{x}}_{d}=\frac{1}{2}\left(\Delta x+\Delta x_{d}\right)$. The properties $\rho =\rho_{n}$ and $c_{p}=c_{p_{n}}$ are evaluated in each time step according to the temperature of the considered CV. The $k_{w}$ and $k_{e}$ represent the thermal conductivities at the interfaces between neighbouring CVs and are calculated from the evaluated thermal conductivities of the considered CV (k(T)) and of the neighbouring CV ($k\left(T_{d}\right)$) in each time step according to Equation 6.

(Equation 6) $$k_{d}=\frac{4k^{\prime }\Delta x}{\Delta x+\Delta x_{d}};k^{\prime }=\frac{k\left(T\right)k\left(T_{d}\right)\left(\Delta x+\Delta x_{d}\right)}{k\left(T_{d}\right)\Delta x+k\left(T\right)\Delta x_{d}+k\left(T\right)k\left(T_{d}\right)R_{d}}\text{,}$$

where $R_{d}$ is the thermal contact resistance at the interface between neighbouring CVs. The code contains an additional iteration loop that corrects the temperatures and conductivities before the time step is continued in order to account for possible numerical instability. This loop is not terminated in the event of numerical instability and issues a warning. Therefore, the user must be careful when choosing the time step, which should be suitable for the expected temperature profiles.

There are four possible boundary conditions to choose from: adiabatic, constant temperature, constant heat flux, and convective. With the adiabatic boundary condition, there is no heat flux through the boundary surfaces of the boundary CVs. With the constant-temperature boundary condition, a constant temperature is defined at the outermost surface of the CVs for the selected boundary. In this case, the conductivity *k* between the surface and the centre point of a boundary CV is determined by the temperature of the boundary CV, with the corresponding distance being half the length of the boundary CV. The boundary condition with a constant heat flux takes into account the defined heat flux at the boundary surface of the boundary CVs. The convective boundary condition takes into account the defined convection coefficient *h*, which determines the heat-transfer rate between the boundary CV and the environment. Together with the heat-transfer coefficient, the outer boundary temperature $T_{b,o}$ is given and the heat flux through the boundary surface is calculated as $h\left(T_{b,o}-T_{b,i}\right)$, where $T_{b,i}$ is the temperature at the inside of the interface, linearly interpolated from the temperatures of the boundary CV and its nearest neighbour, $T_{b,i}=\left(2\Delta x+\Delta x_{d}\right)/\left(\Delta x+\Delta x_{d}\right)T-\Delta x/\left(\Delta x+\Delta x_{d}\right)T_{d}$.

The Equation 5 is written for each CV of the system, resulting in a system of *N* linear equations, where *N* is the discretization number in the *x* direction. The system of linear equations is solved for each successive time step. The matrix of the system of equations is tridiagonal and is therefore solved with an algorithm for tridiagonal matrices.^49^ The number of CVs can be up to 10^6^, but we recommend using up to 10^3^ CVs, as the calculation time increases with the number of CVs. For a basic component made of a material with temperature-dependent properties, a progress of 10 s in real time corresponds to less than one second of computing time if the number of CVs is 10, and the time increases linearly with the number of CVs (assuming good convergence, e.g., continuous material properties). This is due to a base time complexity of O(n) of the tridiagonal matrix algorithm, for continuous material properties. For further analysis of computational time, see supplemental information, Table S1.

#### Numerical model

The numerical model in TCCbuilder is based on a time-dependent, Fourier , heat-transfer equation, with heat conduction as the main heat-transfer mechanism. The model is therefore best suited for modeling solid-state objects, but is also valid in cases when heat conduction is the main heat-transfer mechanism in considered fluids. With the Fourier equation, only macroscopic phenomena are considered, and the lower size limit of the considered part of an object is about 1 μm. The equation is discretized in one dimension (spatial coordinate *x*) according to the finite volume method (FVM), using the backward difference scheme. The scheme is unconditionally stable for continuous functions of material properties. Care should be taken when dealing with discontinuous material properties, such as phase change materials.

In addition to heat transfer, caloric (magnetocaloric, electrocaloric, elastocaloric, and barocaloric) and thermoelectric effects are included in the model. The caloric effects are taken into account via the adiabatic temperature change when an external field (magnetic, electric, stress, or pressure) is applied.

Each TCE or part of a TCE in TCCbuilder is discretized with a finite number of CVs. These parts are connected in series to form a TCC for which a system of equations is created at each time step. By solving the system of equations, the temperatures of all the CVs are updated simultaneously and the time is advanced by one time step.

The source code of the software containing the numerical model is available online as a Github project,^50^ which can be forked and customised to the user’s needs, or where researchers can contribute to the code with additions, changes, improvements or problem reports.

#### Modeling thermal regulator

The evaluation of properties at any time is straightforward for most TCEs. However, for the thermal regulator, more modeling approaches are possible. Here we assume that the regulator consists of a phase-change material and define a way to evaluate its properties during the phase change. The properties and operating characteristics of a thermal regulator in TCCbuilder are defined as follows. The user must specify the temperature range of the phase transition from $T_{1}$ to $T_{2}$, the latent heat *H*, and the base values of the specific heat capacity in both phases (A and B), $c_{p,A},c_{p,B}$. The specified values are used to create the $c_{p}\left(T\right)$ curve^51^ of the regulator according to the Equation 7,

(Equation 7) $$c_{p}=c_{p,A}+\frac{c_{p,A}-c_{p,B}}{1+\exp\;\left(\alpha \left(T_{m}-T\right)\right)}+\frac{\alpha H}{2(1+\cosh\left(\alpha \left(T-T_{m}\right)\right)}\text{,}$$

where $T_{m}=\left(T_{1}+T_{2}\right)/2$ is the mean temperature of the phase transition and α is a parameter that defines the ratio of height to width ratio of the curve, and is chosen as α=5/ΔT, where $\Delta T=\left|T_{2}-T_{1}\right|$. It is assumed that there is no hysteresis. Similarly, the density and thermal conductivity of thermal regulator are calculated using the values for the density and thermal conductivity in both phases, $\rho_{A}$, $\rho_{B}$, and $k_{A}$, $k_{B}$ (specified by the user),

(Equation 8) $$\rho =\rho_{A}+\frac{\rho_{B}-\rho_{A}}{1+\exp\;\left(\alpha \left(T_{m}-T\right)\right)}$$

and

(Equation 9) $$k=k_{A}+\frac{k_{B}-k_{A}}{1+\exp\;\left(\alpha \left(T_{m}-T\right)\right)}\text{.}$$

#### Graphical user interface

The GUI of TCCbuilder was developed as a forked version of an open source circuit simulator by P. Falstad.^52^ It is written in the Java programming language^53^ and is provided online via the Google Web Toolkit.^54^ It currently enables the 1D design of TCEs and TCCs.

In the upper right corner of the GUI, the user should select the dimensionality and the scale before starting the TCC design. The scale determines how much real length can be placed on the canvas. For example, at a scale of 200 μm, a 20 mm TCC will fit on the canvas, and at a scale of 1 mm, a 100 mm TCC will fit on the canvas.

The 1D TCEs can either be designed from scratch with basic components or with templates for TCEs (see Figures 2B and 2C). The construction of the TCEs from basic components gives the user complete freedom of design, i.e., each component of the TCE can be made of a different material or have its own constant properties. On the other hand, the design of TCEs using templates allows a better graphical representation and makes the design of TCCs faster and easier, but the possibilities to specify the properties of parts of TCEs, and the temperature dependence of properites, are limited for some of them. In the case of using TCE templates, the TCEs from the library of TCEs^40^ (which are also available in the GUI) can be integrated into a TCC. Even though the numerical model is the same for both approaches, we do not recommend mixing them for reasons of graphical consistency. The basic components or TCEs are selected from the “Draw” menu and dragged and dropped onto the canvas. The basic components appear as 1D blocks (thick lines), while the TCEs each have their own graphical representation (see Figure 2B) and include thermal conduit, thermal resistor, thermal switch, thermal regulator, thermal diode, thermal capacitor and additionally a heat source and a heat sink (same representation as the heat source). A TCC consists of at least one TCE, but in general a user will design a TCC consisting of several TCEs.

A simulation is initialized by clicking the “Build TCC” button, where the simulation parameters can be set: time step, start temperature of the TCC, the conditions at each boundary (adiabatic, constant heat flux, constant temperature, convective, and periodic), separately, and cyclical operation. The time step can be set between 1 ns and 60 s. The start temperature must be between 0 and 2000 K.

If periodic (cyclical) operation is selected, the user can choose between different parts of the cycle: heat transfer, heat input, mechanical displacement, external field change, property change, temperature change, TCE toggle, time pass, and length change.

As soon as all the simulation parameters have been set, the simulation is started by clicking on the “Apply” button. The course of the temperatures can be displayed either in a diagram in the lower part of the canvas or with the colors of the TCC. The temperature range of the diagram and the color scale can be set manually. All these options are available in the “Options” menu. The progress of the heat-transfer time can be seen in the top left corner of the canvas. The user can stop the simulation at any time by clicking the “Run/STOP” button and export the simulation report (see Data S1). After stopping the simulation, the user can resume it by clicking the “Run/STOP” button.

##### Difference between components and TCEs

The construction of the TCEs from basic components gives the user complete freedom of design, i.e. each component of the TCE can be made of a different material or have its own constant properties. The material for each component is selected from a list of materials in TCCbuilder’s library of materials, accessible from the component’s edit menu. When the mouse pointer is positioned on a material, a tooltip appears with information on the temperature ranges for which the properties of the material are defined. In general, the valid CV temperatures are between 0 and 2000 K. However, if the temperature of a CV is outside the temperature range of the material in which, for example, $c_{p}$ is no longer known, the value for the $c_{p}$ used may be incorrect. When a material is selected for a base component, the properties of that material are read from the material files, which specify the temperature dependence of each property for each CV of the component at each time step. If reading from files is not required, the user can omit this step by checking the “Constant property” checkbox and entering the desired constant value for the selected thermal property. This is possible for all three properties (density, specific heat capacity, and thermal conductivity). Alternatively, the material “Custom” can also be selected, in which case all of the thermal properties for the selected basic component are user-defined and constant. Note that a user who wants to use their own material with temperature-dependent properties can do so by adding the material to the TCCbuilder’s material library and requesting a rebuild of the tool. This adds the material to the material selection in the GUI. Other parameters that can be set are the length of the basic component, the number of CVs, the west and east thermal contact resistance, the heat generation, and the heat loss rate to the ambient. A basic component can have just one CV, but the total number of CVs in the TCC must not be less than three. The length of the individual CVs must exceed 1 μm. With this approach, the TCC essentially consists of basic components connected in series.

On the other hand, the design of TCEs using templates allows a better graphical representation and makes the design of TCCs faster and easier, but the possibilities to specify the properties of parts of TCEs, and the temperature dependence of properites, are limited for some of them (see Table S2). Some functionalities are already implemented, depending on the particular TCE. In the case of a thermal switch, for example, no material can be selected, but two values for the conductivity - for the on or off state, the density and the specific heat capacity of the switch - must be entered. The following TCEs are available: thermal conduit, thermal resistor, thermal switch, thermal regulator, thermal diode, thermal capacitor, and heat source/heat sink. Note that some of these TCEs are essentially basic components with a more specifically descriptive graphical representation. A thermal conduit, for example, is technically no different from a basic component, but its thermal conductivity is usually set to a high value (> 1 Wm^–1^ K^−1^) or, alternatively, a thermally conductive material is chosen for a conduit. In other words, there is no restriction on setting the properties of a conduit, just as with the basic component.

##### Boundary conditions

In the case of selected constant temperature, convective, or periodic boundary condition, the boundary temperature must be specified. If the convective boundary condition is selected, the convection coefficient must also be specified. The periodic boundary condition is essentially the same as the constant temperature boundary condition, only with a sinusoidal change in the boundary temperature. In this case, the mean temperature, the amplitude of the thermal oscillation, and the frequency of the thermal oscillation must be specified.

##### Possible cycle parts in cyclic operation of a TCC

(1) The heat-transfer cycle part advances the time by one time step until the duration of this part (specified by the user) is reached. This means that the heat is transferred without any special features, in accordance with the heat-transfer equation and the specified boundary conditions.
(2) The heat input cycle part allows the user to set the heat generation in selected basic components or TCEs for a limited period of time in a cycle.
(3) The mechanical displacement is currently an instantaneous event which allows a change in the composition of the device in such a way that the order of the components or TCEs in a TCC is changed. Each basic component or TCE that changes its position in the TCC must be selected and a new index for the component/TCE must be entered.
(4) The external field change is currently an instantaneous event in which the external field is switched on or off for selected basic components or TCEs, depending on its current state. Only basic components or TCEs made of caloric material can be selected in this part of the cycle. The magnitude of the external field must be selected from the drop-down list and the external field is initially switched off (this can be changed in the Edit dialog of a basic component or a TCE).
(5) The properties change cycle part allows the user to periodically change one or more properties of selected basic components. If the duration of this part of the cycle is set to $t_{\text{cycle}\;\text{part}}>0$, the heat-transfer in this part of the cycle takes place simultaneously and the value of each selected property changes linearly with time from the start value to the end value.
(6) The temperature change cycle part allows the user to periodically change the temperature of selected basic components. If the duration of this cycle part is set to $t_{\text{cycle}\;\text{part}}>0$, the heat-transfer in this cycle part takes place simultaneously and the temperature changes linearly with time from the start value to the end value.
(7) The TCE toggle cycle part instantaneously switches the selected TCEs. Only TCEs that can be toggled (switched on and off; currently, the only such TCE is the thermal switch) can be added to this cycle part. Each time this cycle part is executed, the selected TCEs are switched on or off depending on their current state.
(8) The time pass cycle part advances the time for the specified amount of time without any heat-transfer or other action. This is usually used after one of instantaneous events, to allow for the the time delay, e.g. of mechanical displacement.
(9) The length change cycle part changes the length of selected components or TCEs.

##### Simulation report

The report contains the simulation parameters - time step and boundary conditions, description of the TCC with listed names and properties of basic components or TCEs, and the temporal development of the temperatures of all CVs. It also includes the heat fluxes at the boundaries of each CV, the average thermal diffusivities of basic components or TCEs, the basic assessment of the adequacy of the space discretization, the heat fluxes at the western and eastern boundaries of the TCC, and actuation power inputs for each TCE, all calculated at the time the report was written. The report can include the temperatures at each time step or at a specific user-defined interval (see also Data S1).

#### Modeling examples details

##### Example 1: Running a simulation of a solid-state thermal diode

The basic components for both parts of the LSCO-LCO thermal diode are drawn by dragging and dropping from the Draw menu in the top left corner. The parameters of each basic component are edited by right-clicking on the component and selecting the “Edit” option. The materials LSCO and LCO are selected for the two components so that the properties of both materials are read from the TCCbuilder’s library of materials. Once the diode has been drawn, the user can set the parameters for the simulation by clicking on buildTCC in the top right corner. Clicking on “Apply” will start the simulation. If the user wants to export the results, they can do so by clicking on “Export Report As Text” or “Save Report As” in the file menu. The report includes the values of the heat fluxes between the CVs (see Data S1).

##### Example 2: Modeling transient operation of a TCC in electronics

Although it is difficult to predict 2D heat-transfer effects in a 1D simulation, some assumptions can be made to qualitatively model the TCC with TCCbuilder. First, the heat-transfer through the system in Figure 3A is mainly 1D in vertical direction, as the GaN device generates 5 W heat that is dissipated downward and mainly to the top of the system, where convection is much larger than at the sides of the system (natural convection). Second, most of the transverse heat-transfer occurs by natural convection through the walls of the thermal buffer.

Figure 3C shows the design of the corresponding TCC in TCCbuilder. The TCC is drawn horizontally, rotated by 90° clockwise according to Figure 3B. The PCB is modelled as a resistor with five CVs, the GaN device is modelled as a capacitor with one CV, the thermal buffer is modelled as a capacitor with 10 CVs, and the spreader base and top are modelled as thermal conduits with five CVs each. The time step in the simulation was Δt= 0.01 s.

The PCB is modelled so that its thermal resistance corresponds to the thermal resistance to the underside of the device in,^43^ which is $R_{B}=$ 65 KW^–1^. The length of the PCB is chosen as $l_{PCB}=$ 2 mm and the thermal conductivity is calculated as $k_{PCB}=$ 0.1 Wm^–1^ K^–1^. A constant temperature of 295 K is used on the underside of the PCB. The density and specific heat capacity of the PCB are estimated taking into account the materials from which PCBs are usually made ^55^ and are assumed to be ρ= 2000 kgm^–3^ for the density and $c_{p}=$ 360 Jkg^–1^ K^–1^ for the specific heat capacity. The GaN device in our model is 0.7 mm thick according to the manufacturer’s data sheet.^56^ The volumetric heat generation is calculated for the GaN and is ${\dot{q}}_{\text{gen}}=$ 2.8 × 10^7^ Wm^–3^. The density, specific heat capacity, and thermal conductivity of gallium nitride are taken from.^57^ The density is ρ= 6150 kgm^–3^, the specific heat capacity is $c_{p}=$ 490 Jkg^–1^ K^–1^ and the thermal conductivity is 130 Wm^–1^ K^–1^. The thermal resistance between the GaN and the first (bottom) heat spreader in^43^ was between 1.8 KW^–1^ and 2.6 KW^–1^, and we chose the highest value because the 1D simulation overestimates the thermal contact between the GaN device and the bottom spreader. The bottom spreader is modelled as a block instead of a pyramid shape, with a thickness of 2.6 mm. It is made of copper with a density of $\rho_{\text{Cu}}=$ 8960 kgm^–3^, a specific heat capacity of $c_{p,\text{Cu}}=$ 385 Jkg^–1^ K^–1^ and a thermal conductivity of $k_{\text{Cu}}=$ 360 Wm^–1^ K^–1^. The thickness of the thermal buffer is 5.6 mm and in our simulation consists exclusively of a PCM with a density of $\rho_{\text{PCM}}=$ 7922 kgm^–3^, a specific heat capacity of $c_{p,\text{PCM}}=$ 290 Jkg^–1^ K^–1^, a thermal conductivity of $k_{\text{PCM}}=$ 360 Wm^–1^ K^–1^ and a latent heat of H= 38 kJkg^–1^.^43^ The top spreader with fins is modelled in the same way as base spreader, with a thickness of 2.6 mm, and the convection through the fins is modelled using an effective convection coefficient. The convection coefficient from the surface with the fins to the airflow was $h_{\text{fins}}=$ 85 Wm^–2^ K^–1^.^43^ Since the surface of the top spreader in TCCbuilder is flat, an effective convection coefficient is used, $h_{\text{eff}}=h_{\text{fins}}\times A_{\text{fins}}/A=$ 270 Wm^–2^ K^–1^. The volumetric heat loss through the sides of the thermal buffer and through the top spreader is set to $h_{V}=h_{\text{nat}}\times P/A=$ 2000 Wm^–3^ K^–1^), where *P* is the perimeter of the buffer and the spreader and $h_{\text{nat}}=$ 8 Wm^–2^ K^–1^ is assumed for the natural convection to the ambient air.

##### Example 3: Modeling of a double-unit electrocaloric cooling device

In the equivalent 1D TCC in TCCbuilder (Figure 4B), the PDMS spacers are not considered. They do not have a large impact on the heat-transfer through the device as we consider the transfer in the vertical direction through the centre of the device. The lengths of the basic components were chosen according to.^44^ The air gap was modelled with a length of 420 μm instead of 3 mm to fit the circuit on the canvas. For this reason, the thermal conductivity of air was changed from 0.2587 Wm^–1^ K^–1^ to 0.0362 Wm^–1^ K^–1^ to obtain the same thermal resistance. The thermal mass of air does not considerably affect the operation of the device. The adiabatic temperature change of the polymers resulting from the EC effect was taken from^44^ and is 2.8 K at an applied electric field of 60.6 MVm^–1^ at room temperature. Due to the limited data on the entropy changes of caloric materials, the adiabatic temperature change in TCCbuilder is modeled as an instantaneous event. The temperature of each affected control volume (CV) changes by $\Delta T_{\text{ad}}$, which depends on the temperature of the CV and the strength of the applied external field, and is provided in the TCCbuilder’s library of materials for selected materials. The movement of the EC polymers in TCCbuilder was simulated by the mechanical displacement that changes the positions of the polymers and the air gaps in each part of the device.

Table S3 shows the parameters of the components used in the TCCbuilder simulation. The operating frequency is 1 Hz, where the time for both heat-transfer processes is 0.45 s, and the time for changing of EC polymers positions is 0.05 s.^44^

The thermal contact resistance was not measured in the experiment. The contact between the EC polymer and other parts of the device (PET and air) is most likely not ideal. We estimate that the thermal contact resistance *R* is less than 10^–2^ m^2^ KW^–1^.^36^^,^^58^^,^^59^ Simulations show that R< 10^–4^ m^2^ KW^–1^ has a negligible influence on the temporal development of device temperature and that there is no major difference between R= 10^–4^ m^2^ KW^–1^ and R= 10^–3^ m^2^ KW^–1^. Therefore, in each simulation, the thermal contact resistance between all components (except between PI and PET films, where the thermal resistance is assumed to be negligible) was set to one of the respective values: 10^–4^, 5 × 10^–3^, or 10^–2^ m^2^ KW^–1^.

We used convective boundary conditions at both ends of the device in our simulations. The convection coefficient was not measured in the experiment, but, there was no forced convection. This indicates that natural convection occurs at both ends of the device as well as at the sides of the device. The latter is accounted for in our simulations as volumetric heat loss or gain. The ambient temperature was set to 298.15 K. In each simulation, the value of *h* was set to one of the respective values: 5, 10 or 25 Wm^–2^ K^–1^.

##### Example 4: TCCbuilder validation with a TCC comprising a heat engine

The dimensionless quantities as well as all the parameters of the simulation are described in the supplemental information (Table S4).

#### Note about a parallel model

The TCCbuilder currently allows TCEs to be connected in series. If the TCEs are connected in parallel, there are more possibilities for modeling. One possibility is to use the heat conduction equation in 2D. The TCCbuilder contains a 2D model, but the tool running online is unfortunately not able to handle the complexity of the calculations. The other option is to consider TCEs like electrical elements, specify their capacitance and resistance and solve the equations resulting from Kirchhoff’s laws in the junctions of the thermal circuits.^60^ This would be a separate model in the tool and is part of the planned future upgrades.

## Supplemental information

Document S1. Tables S1–S4

Data S1. Simulation report

## Footnotes

Supplemental information can be found online at https://doi.org/10.1016/j.isci.2024.111263.
