# Supplementary material for: TCCbuilder: An open-source tool for the analysis of thermal switches, thermal diodes, thermal regulators, and thermal control circuits
Source: iScience. 2024 Oct 28;27(12):111263. doi: 10.1016/j.isci.2024.111263 (PMC11700648; doi:10.1016/j.isci.2024.111263)
Supplement: Document S1. Tables S1–S4 [file mmc1.pdf]

iScience, Volume *27*

Supplemental information

TCCbuilder: An open-source tool for the analysis of thermal switches, thermal diodes, thermal regulators, and thermal control circuits

Katja Vozel, Katja Klinar, Nada Petelin, and Andrej Kitanovski

Supplemental information

Contents

Computational complexity

Graphical user interface and model details
Difference between Components and TCEs . . . .

Modeling examples details
Example 3: modeling of a double-unit electrocaloric cooling device . . . .
Example 4: TCCbuilder validation with a TCC comprising a heat engine . . . .

List of Tables

S1 Examples of computation times in TCCbuilder tool. . . . .
S2 Preset functionalities (restrictions) of the TCE templates in TCCbuilder. . . . .
S3 Parameters of the basic components used in the TCCbuilder simulation of the double-unit electrocaloric cooling device. . . . .
S4 Variables, parameters, their meaning, and their values in simulations of a TCC comprising a heat engine. . . . .

Computational complexity

Some examples of calculation times are given in Table S1, to give the reader an idea of how much time is needed to run the simulations. The equivalent computation time τ corresponds to one second of real time.

Table S1: **Examples of computation times in TCCbuilder tool.**

TCE/TCC	Number of CVs	Time step (ms)	τ (ms)
Component	10	10	80
Component	100	10	760
Component	10	0.1	8300
GaN device circuit [1]	26	100	170

Graphical user interface and model details

Difference between Components and TCEs

Table S2: **Preset functionalities (restrictions) of the TCE templates in TCCbuilder.**

TCE	Restrictions
Thermal Conduit	/
Thermal Resistor	/
Thermal Switch	material is not specified, ρ, c_p, and $k_{\text{on}}/k_{\text{off}}$ values must be input as constant values
Thermal Regulator	material is not specified, ρ, c_p, and k values must be input for both phases, temperature range of phase change, and latent heat of phase change, all as constant values
Thermal Diode	material is not specified, ρ, c_p, and $k_{\text{forward}}/k_{\text{backward}}$ values must be input as constant values
Thermal Capacitor	/
Heat Source/Sink	/

Modeling examples details

Example 3: modeling of a double-unit electrocaloric cooling device

Table S3 shows the parameters of the components used in the TCCbuilder simulation. The operating frequency is 1 Hz, where the time for both heat-transfer processes is 0.45 s, and the time for changing of EC polymers positions is 0.05 s [2].

Table S3: **Parameters of the basic components used in the TCCbuilder simulation of the double-unit electrocaloric cooling device.**

Basic component	length (mm)	ρ (kgm^{-3})	c_p (Jkg^{-1}K^{-1})	k (Wm^{-1}K^{-1})
PI film 1,2	0.05	1420	1090	0.8
PET1,3	0.05	1380	2150	0.19
PET 2	0.1	1380	2150	0.19
EC 1,2	0.07	2100	1420	0.32
Air gap 1,2	0.42	1.2	1006	0.036

Example 4: TCCbuilder validation with a TCC comprising a heat engine

The parameters, that are relevant for the modeling of the temperature difference across the TE engine and of its power density, are described in Table S4. The values of parameters used for both simulations (Figure 6B and Figure 6C) are also listed in the table.

Table S4: **Variables, parameters, their meaning, and their values in simulations of a TCC comprising a heat engine.**

Var./par.	Meaning	Units	Value Figure 6B	Value Figure 6C
L_1	Length of thermal mass	m	0.01	0.01
L_{d}	Length of thermal diode	m	0.01	0.01
L_{TE}	Length of TE engine	m	0.01	0.01
ρ_1	Density of thermal mass	kgm^{-3}	5000	2000
ρ_{d}	Density of thermal diode	kgm^{-3}	5	5
ρ_{TE}	Density of TE engine	kgm^{-3}	5	5
c_{p1}	Specific heat capacity of thermal mass	Jkg^{-1}K^{-1}	300	10,000
$c_{p\text{d}}$	Specific heat capacity of thermal diode	Jkg^{-1}K^{-1}	5	5
$c_{p\text{TE}}$	Specific heat capacity of TE engine	Jkg^{-1}K^{-1}	5	5
k_1	Thermal conductivity of thermal mass	Wm^{-1}K^{-1}	1250	1000
$k_{\text{d,0}}$	Base thermal conductivity of a diode	Wm^{-1}K^{-1}	1250	1000
R_{TE}	Thermal resistivity of TE engine	m^{2}KW^{-1}	0.008	0.008
α_1	Thermal diffusivity of thermal mass	m^{2}s^{-1}		
β	Level of thermal rectification	/	0.7, 0.95	0.99
$\gamma' = \gamma T_A$	Dimensionless steepness of thermal rectification	/	10^4	10^4
T_0	Mean ambient temperature	K	293	293
T_A	Amplitude of temperature fluctuation	K	50	10
$\tau = t\frac{\alpha_1}{L_1^2}$	Dimensionless time	/	/	/
ω	Frequency of temperature fluctuation	s^{-1}	13.20	/
$\nu = \omega\frac{L_1^2}{\alpha_1}$	Dimensionless frequency of temperature fluctuation	/	10$^{0.2}$	/
$Bi_{\text{TE}} = \frac{L_1}{k_1 R_{\text{TE}}}$	Thermal resistance ratio of linear thermal masses to TE engine	/	0.001	0.001

Supplemental references

[1] T. Yang, P. V. Braun, N. Miljkovic, W. P. King, Phase change material heat sink for transient cooling of high-power devices, Int. J. Heat Mass Transf. 170 (2021) 121033. doi:https://doi.org/10.1016/j.ijheatmasstransfer.2021.121033.

[2] Y. Bo, Q. Zhang, H. Cui, M. Wang, C. Zhang, W. He, X. Fan, Y. Lv, X. Fu, J. Liang, Y. Huang, R. Ma, Y. Chen, Electrostatic actuating double-unit electrocaloric cooling device with high efficiency, Adv. Energy Mater. 11 (13) (2021) 2003771. doi:https://doi.org/10.1002/aenm.202003771.
